# Structural Reconfiguration of the Time-Valid Cohort and Stage-Specific Reversal of Prehospital Time–Outcome Associations During the COVID-19 Pandemic

**DOI:** 10.3390/medicina62020302

**Published:** 2026-02-02

**Authors:** Chiwon Ahn, Jae Hwan Kim, Young Taeck Oh

**Affiliations:** Department of Emergency Medicine, College of Medicine, Chung-Ang University, Seoul 06973, Republic of Korea; cahn@cau.ac.kr (C.A.); sult@cau.ac.kr (J.H.K.)

**Keywords:** out-of-hospital cardiac arrest (OHCA), emergency medical services (EMS), pandemics, neurological outcome

## Abstract

*Background and Objectives*: During the COVID-19 pandemic, worsening outcomes after out-of-hospital cardiac arrest (OHCA) have been widely reported and are often attributed to prolonged prehospital system delays. However, little attention has been paid to whether the population of patients with analyzable prehospital time data—the time-valid cohort—itself changed during the pandemic, or to how such changes may have influenced the observed association between prehospital time and outcomes. To examine structural changes in the time-valid OHCA cohort across pandemic phases, and to evaluate phase-specific associations between call-to-emergency department (ER) time and neurological outcomes. *Materials and Methods*: We conducted a nationwide retrospective observational study using a Korean OHCA registry from 2016 to 2022 (*n* = 217,356). Patients with logically consistent prehospital time intervals from arrest recognition to ER arrival were defined as the time-valid cohort (*n* = 62,240). Pandemic phases were categorized as pre-pandemic (2016–2019), early pandemic (2020), and prolonged pandemic (2021–2022). Changes in cohort composition were assessed descriptively and visually. Associations between call-to-ER time (per 10-min increase) and good neurological outcome (Cerebral Performance Category 1–2) were evaluated using phase-stratified logistic regression models adjusted for age, sex, and initial rhythm. *Results*: The time-valid cohort consisted exclusively of witnessed arrests throughout the study period. As the pandemic progressed, the cohort became older (median age increased from 70 to 72 years), and the proportion of shockable rhythm declined from 21.7% in the pre-pandemic period to 17.5% in the prolonged pandemic period. The proportion of good neurological outcomes decreased from 9.3% to 6.8%. Before the pandemic, longer call-to-ER time was associated with a lower likelihood of a good neurological outcome (odds ratio [OR] per 10-min increase, 0.85; 95% confidence interval [CI], 0.83–0.88). This association was attenuated during the early pandemic phase (OR, 0.95; 95% CI, 0.91–1.00) and reversed during the prolonged pandemic phase (OR, 1.07; 95% CI, 1.04–1.10). *Conclusions*: Changes in the association between prehospital time and neurological outcome during the COVID-19 pandemic cannot be interpreted as the effect of system delay alone. Instead, these findings should be understood in the context of substantial structural reconfiguration of the time-valid OHCA cohort, which became progressively older and physiologically less favorable across the pandemic’s phases. Consideration of cohort structure is essential when interpreting prehospital time–outcome relationships during large-scale system disruptions.

## 1. Introduction

Out-of-hospital cardiac arrest (OHCA) is associated with high mortality and poor neurological outcomes worldwide [1,2,3]. Despite advances in emergency medical service (EMS) systems and post–cardiac arrest care, overall survival rates remain low, and neurological impairment continues to be common among survivors. Prehospital time intervals—particularly the time from arrest recognition to hospital arrival—have therefore been regarded as critical determinants of outcome and have been extensively studied as modifiable system-level factors [4,5,6].

Since the onset of the coronavirus disease 2019 (COVID-19) pandemic, numerous studies have reported significant changes in the incidence, characteristics, and outcomes of OHCA across different regions [7,8,9,10]. In Republic of Korea and other countries with well-established EMS systems, these changes have generally included increased EMS response and transport times, reduced bystander cardiopulmonary resuscitation (CPR), a lower proportion of shockable rhythms, and worsened survival or neurological outcomes [11,12,13,14]. Consequently, much of the existing literature has interpreted pandemic-related outcome deterioration primarily as a consequence of system delays and disruptions in prehospital care.

However, an important methodological assumption underlies most prehospital time–outcome analyses: that the population of patients with available and analyzable prehospital time data is stable over time. In practice, meaningful analysis of prehospital time intervals requires that arrest recognition time, EMS activation, and hospital arrival times are clearly documented and logically consistent. Patients for whom these conditions are not met are necessarily excluded from time-based analyses. This creates a subset of patients—herein defined as the “time-valid cohort”—that is often treated as a neutral analytic denominator rather than as a potentially selected cohort with its own structural characteristics. Importantly, the exclusion of cases with missing or implausible time data is not merely a passive data-cleaning step but an active selection process. Since data missingness often correlates with chaotic resuscitation environments or early futility, excluding these cases systematically reconfigures the study population, introducing inherent selection bias [15]. Whether this time-valid cohort itself changed during the COVID-19 pandemic has not been systematically examined. Large-scale system disruptions, such as pandemics, can influence not only EMS performance but also bystander behavior, transport decisions, documentation practices, and the physiological state of patients who ultimately reach the hospital. These factors may selectively shape which patients have complete and reliable time records, thereby altering the composition of the cohort used for time–outcome analyses. If such structural changes occur, the observed associations between prehospital time and outcome may reflect cohort reconfiguration rather than direct effects of time delay.

Existing pandemic-related OHCA studies have largely focused on comparing outcomes before and during the pandemic or quantifying changes in specific system metrics, such as response time or transport duration [7,8,9,10,11,12,13,14]. While these approaches are informative, they do not explicitly address whether the analytic population for time-based outcomes remains comparable across periods. In particular, few studies have examined whether patients with analyzable prehospital time intervals differ systematically across pandemic phases in terms of age distribution, initial rhythm, or neurological prognosis.

We hypothesized that the COVID-19 pandemic not only affected EMS system performance but also reshaped the population of OHCA patients for whom prehospital time intervals could be reliably analyzed. Specifically, we postulated that the time-valid cohort underwent structural reconfiguration during the pandemic and that this reconfiguration influenced the observed association between prehospital time and neurological outcome.

Therefore, the objectives of this study were twofold. First, we sought to characterize structural changes in the time-valid OHCA cohort across distinct pandemic phases, focusing on patient demographics, arrest characteristics, and outcomes. Second, we aimed to evaluate whether the association between call-to-emergency department (ER) time and neurological outcome differed across the pandemic’s phases, and to interpret any observed changes within the context of cohort reconfiguration rather than as isolated effects of system delay.

## 2. Materials and Methods

### 2.1. Study Design and Data Source

**Figure 1:**
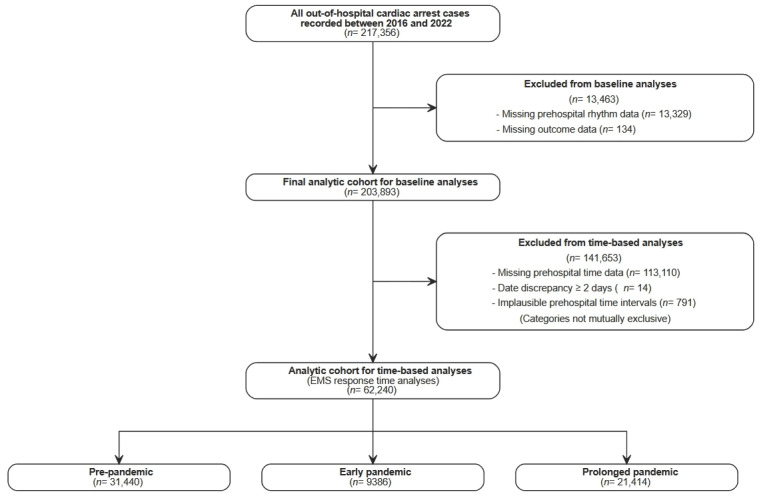
Flow diagram of cohort selection and derivation of the analytic cohort for time-based analyses (the time-valid cohort).

This study was a nationwide retrospective observational analysis using data from the Out-of-Hospital Cardiac Arrest Surveillance (OHCAS) database, managed by the Korea Disease Control and Prevention Agency (KDCA). The detailed selection process and derivation of the time-valid cohort are illustrated in Figure 1. The registry prospectively collects standardized data on OHCA events attended by emergency medical services (EMS), including patient demographics, arrest circumstances, prehospital interventions, time stamps, and hospital outcomes. Data are recorded using uniform definitions and quality-control procedures across participating regions.

All OHCA cases recorded between 1 January 2016 and 31 December 2022 were screened for eligibility. This study was designed and reported in accordance with the Strengthening the Reporting of Observational Studies in Epidemiology (STROBE) guidelines for observational research [16].

### 2.2. Study Population

A total of 217,356 OHCA cases were initially identified from the registry. We first constructed a baseline analytic cohort by excluding patients with missing prehospital rhythm data or missing outcome information. This baseline cohort was used to describe overall patient characteristics across pandemic phases.

For analyses involving prehospital time intervals, we further defined a time-valid cohort. Inclusion in the time-valid cohort required that prehospital time information satisfied all of the following criteria:

A documented date and time of arrest recognition;
A documented date and time of EMS call receipt;
A documented date and time of emergency department (ER) arrival;
Logical consistency of time intervals, defined as non-negative intervals without excessive duration or implausible date discrepancies.

Cases with negative time intervals, extreme values exceeding predefined thresholds, or date mismatches suggestive of recording errors were excluded from the time-based analyses. These criteria were applied to minimize misclassification and ensure analytic validity of prehospital time measurements.

### 2.3. Definition of Pandemic Phases

Pandemic phases were defined a priori based on calendar year to reflect major shifts in healthcare system strain and societal response:

- Pre-pandemic period: 2016–2019;
- Early pandemic period: 2020;
- Prolonged pandemic period: 2021–2022;

This categorization was intended to distinguish the acute disruption phase following the emergence of COVID-19 from the subsequent period characterized by partial system adaptation and sustained pandemic conditions. Specifically, these phases align with major shifts in Republic of Korea’s national quarantine policies (ranging from intensified social distancing to the “Living with COVID-19” scheme) and the prevalence of dominant viral variants (e.g., the Delta variant in 2021 and Omicron in 2022), which imposed distinct burdens on the emergency care system.

### 2.4. Variables and Definitions

#### 2.4.1. Exposure Variable

The primary exposure of interest was call-to-ER time, defined as the interval between EMS call receipt and arrival at the emergency department. This interval was analyzed as a continuous variable and scaled per 10-min increase to facilitate clinical interpretability.

#### 2.4.2. Outcome Variable

The primary outcome was good neurological outcome, defined as a Cerebral Performance Category (CPC) score of 1 or 2 at hospital discharge. Neurological outcomes were assessed according to standard registry definitions.

#### 2.4.3. Covariates

Covariates were selected based on prior literature and clinical relevance to OHCA outcomes [1,2,3,4,5,6]. These included the following:

- Age, treated as a continuous variable;
- Sex (male or female);
- Initial cardiac rhythm, categorized as shockable (ventricular fibrillation or pulseless ventricular tachycardia) or non-shockable (asystole or pulseless electrical activity).

Although other variables such as bystander CPR and comorbidities were described in the baseline analyses, covariate adjustment in time–outcome models was intentionally limited to preserve model stability and avoid overadjustment, given this study’s focus on structural interpretation rather than causal estimation.

### 2.5. Statistical Analysis

The baseline characteristics of the analytic cohort were summarized using medians with interquartile ranges (IQRs) for continuous variables and counts with percentages for categorical variables. The characteristics of the time-valid cohort were summarized in the same manner to describe its composition across pandemic phases.

To evaluate associations between call-to-emergency department time and neurological outcome, multivariable logistic regression models were constructed and stratified by pandemic phase. Separate models were fitted for the pre-pandemic, early pandemic, and prolonged pandemic periods. Results were reported as odds ratios (ORs) with 95% confidence intervals (CIs).

Model-based predicted probabilities of good neurological outcome were estimated across the observed range of call-to-emergency department time, holding covariates at reference values, to support interpretation of the regression results.

Several sensitivity analyses were performed. First, restricted cubic spline models were used to assess potential non-linearity in the time–outcome relationship. To minimize selection bias and avoid overfitting, we utilized a pre-specified model structure with three knots placed at standard recommended percentiles (the 10th, 50th, and 90th percentiles) of the call-to-ER time distribution [17]. Second, the stability of linear estimates was evaluated by excluding extreme call-to-emergency department times at various trimming thresholds. Third, inverse probability weighting was applied to account for differential inclusion into the time-valid cohort, with weights derived from a logistic regression model predicting time-valid status based on available baseline characteristics.

All analyses were performed using R software (version 4.3). Statistical significance was assessed using a two-sided alpha level of 0.05. Given the observational design, findings were interpreted as associations rather than causal effects.

### 2.6. Ethical Considerations

The study protocol was reviewed by the Institutional Review Board of Chung-Ang University Hospital and was determined to be exempt from review (IRB No. 2505-018-19576). The requirement for informed consent was waived due to the retrospective nature of the study and the use of de-identified data.

### 2.7. Use of Artificial Intelligence

Generative artificial intelligence (Claude 4.5 Opus, Anthropic, San Francisco, CA, USA) was used to assist with manuscript editing and visual abstract creation. All AI-generated outputs were critically reviewed, verified for accuracy, and revised as necessary by the authors, who take full responsibility for the final content.

## 3. Results

### 3.1. Study Population and Cohort Selection

A total of 217,356 out-of-hospital cardiac arrest (OHCA) cases recorded between 2016 and 2022 were initially screened (Figure 1). After exclusion of patients with missing prehospital rhythm data or missing outcome information, 203,893 patients constituted the baseline analytic cohort.

For analyses involving prehospital time intervals, additional exclusions were applied to ensure logical consistency and plausibility of the time data. Specifically, cases with missing arrest recognition time, EMS call receipt time, or emergency department (ER) arrival time were excluded, as were cases with negative time intervals, excessive durations, or date discrepancies. Following these exclusions, 62,240 patients comprised the final time-valid cohort used for prehospital time–outcome analyses.

Importantly, all patients in the time-valid cohort had a clearly documented arrest recognition time, resulting in a witnessed arrest proportion of 100% across all pandemic phases.

The final analytic cohort was further stratified by pandemic phase for regression analyses.

### 3.2. Baseline Characteristics Across Pandemic Phases

The baseline characteristics of the analytic cohort stratified by pandemic phase are summarized in Table 1. The median age of patients increased progressively across the study periods, from 69 years (interquartile range [IQR], 55–80) in the pre-pandemic period to 71 years (IQR, 57–82) in the prolonged pandemic period. The proportion of male patients remained relatively stable across phases.

The proportion of arrests occurring in public locations declined over time, while the proportion of medical causes increased during the pandemic. Comorbid conditions, including hypertension, diabetes mellitus, and pre-existing heart disease, became more prevalent in later periods.

Across pandemic phases, the proportion of shockable initial rhythms decreased, from 13.1% in the pre-pandemic period to 11.1% during the prolonged pandemic period. Correspondingly, survival to hospital discharge and good neurological outcomes declined, with good neurological outcome decreasing from 5.0% before the pandemic to 4.2% during the prolonged pandemic period.

Values are presented as the median (interquartile range) for continuous variables and number (percentage) for categorical variables. † Age was compared using the Kruskal–Wallis test. All other categorical variables were compared using the chi-squared test. The pandemic phases were defined as pre-pandemic (2016–2019), early pandemic (2020), and prolonged pandemic (2021–2022). Given the large sample size, statistical significance should be interpreted in the context of clinical relevance.

### 3.3. Structural Changes in the Time-Valid Cohort

**Figure 2:**
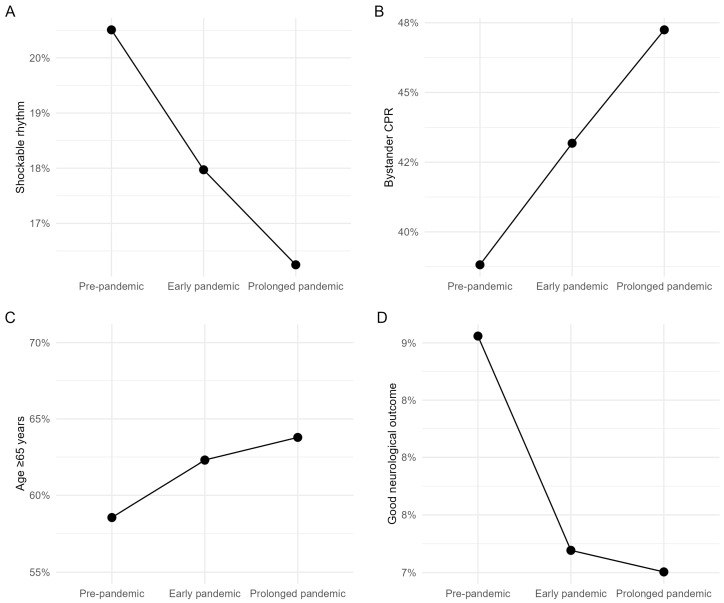
Structural changes in the strict time-valid cohort (*n* = 62,240) across pandemic phases: (**A**) Proportion of shockable initial rhythm. (**B**) Proportion of bystander cardiopulmonary resuscitation. (**C**) Proportion of patients aged ≥65 years. (**D**) Proportion of good neurological outcomes (CPC 1–2).

The characteristics of the time-valid cohort stratified by pandemic phase, are presented in Table 2 and visualized in Figure 2. Structural changes in the time-valid cohort across pandemic phases are illustrated in Figure 2. Despite restriction to witnessed arrests, the composition of the time-valid cohort shifted substantially over time, with progressive aging of patients, a declining proportion of shockable rhythm, and a reduced proportion of good neurological outcomes, alongside an increase in bystander cardiopulmonary resuscitation. Within this cohort, the median age increased from 70 years (IQR, 55–80) in the pre-pandemic period to 72 years (IQR, 58–82) during the prolonged pandemic period.

The proportion of shockable initial rhythms declined steadily across phases, from 21.7% in the pre-pandemic period to 18.8% in the early pandemic and 17.5% in the prolonged pandemic period. Although the proportion of bystander cardiopulmonary resuscitation increased over time, from 32.0% before the pandemic to 37.0% during the prolonged pandemic period, this increase did not translate into improved neurological outcomes.

The proportion of patients achieving a good neurological outcome decreased from 9.3% in the pre-pandemic period to 7.2% during the early pandemic and further to 6.8% during the prolonged pandemic period.

These trends were observed despite restriction to witnessed arrests, indicating that structural changes in the time-valid cohort were not attributable to differences in arrest observability but rather reflected shifts in patient vulnerability and early physiological status.

Values are presented as the median [interquartile range] or number (%). The strict time-valid cohort included patients with logically consistent arrest-to-call, call-to-emergency department, and arrest-to-emergency department time intervals and complete neurological outcome data. Abbreviations: CPR, cardiopulmonary resuscitation; ROSC, return of spontaneous circulation; CPC, Cerebral Performance Category.

### 3.4. Pandemic Phase–Specific Associations Between Call-to-ER Time and Neurological Outcome

**Figure 3:**
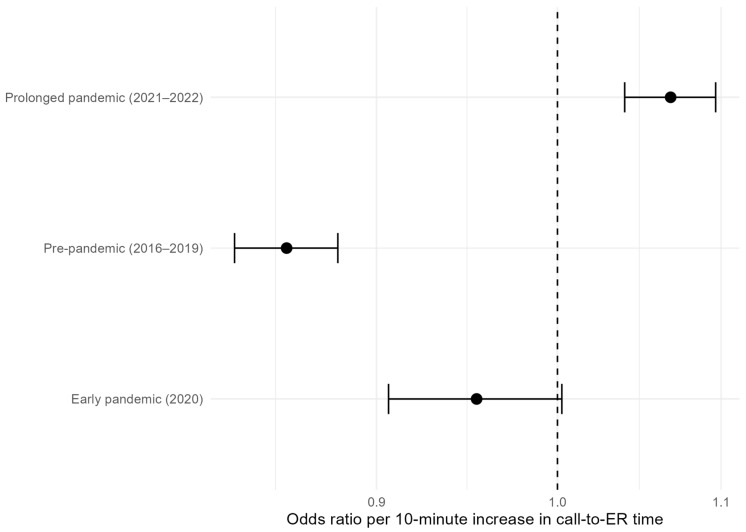
Phase-specific associations between call-to-emergency department time and neurological outcome.

Associations between call-to-emergency department time and good neurological outcome stratified by pandemic phase are summarized in Table 3 and visualized in Figure 3.

In the pre-pandemic period, a longer call-to-emergency department time was significantly associated with a lower likelihood of a good neurological outcome (odds ratio [OR] per 10-min increase, 0.85; 95% confidence interval [CI], 0.83–0.88). During the early pandemic period, this association was attenuated and was no longer statistically significant (OR, 0.95; 95% CI, 0.91–1.00). In contrast, during the prolonged pandemic period, a longer call-to-emergency department time was positively associated with good neurological outcome (OR, 1.07; 95% CI, 1.04–1.10).

**Figure 4:**
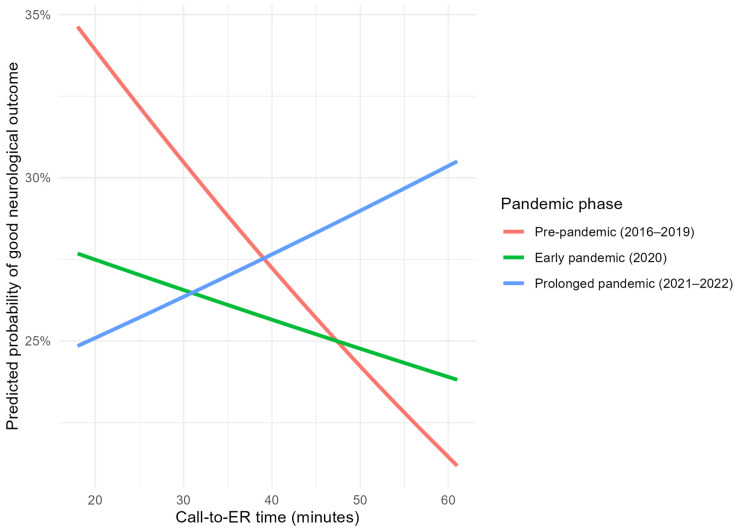
Model-based predicted probabilities of good neurological outcome across pandemic phases.

Model-based predicted probability curves further illustrate phase-specific changes in the time–outcome relationship, demonstrating progressive attenuation and flattening of the association during the early pandemic phase and an apparent inversion during the prolonged pandemic phase (Figure 4).

Odds ratios represent the change in the odds of a good neurological outcome (CPC 1–2) per 10-min increase in call-to-emergency department time. Models were adjusted for age, sex, and initial rhythm and were fitted separately for each pandemic phase. *p* values < 0.05 were considered statistically significant.

Predicted probabilities of good neurological outcome (CPC 1–2) are shown across call-to-emergency department time based on phase-stratified logistic regression models, with covariates held at reference values. Curves illustrate phase-specific changes in the time–outcome relationship.

Odds ratios (ORs) and 95% confidence intervals represent the change in the odds of a good neurological outcome (CPC 1–2) per 10 -min increase in call-to-emergency department time, as estimated from multivariable logistic regression models stratified by pandemic phase. The models were adjusted for age, sex, and initial rhythm.

### 3.5. Sensitivity Analyses

Several sensitivity analyses were performed to evaluate the robustness and interpretability of the observed association between call-to-emergency department time and neurological outcome.

**Figure 5:**
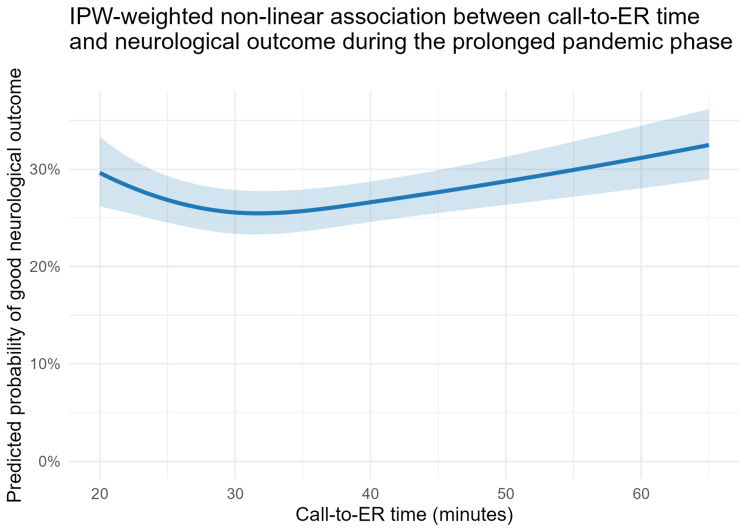
IPW-weighted spline-based association between call-to-emergency department time and neurological outcome during the prolonged pandemic phase.

First, restricted cubic spline models were applied to assess potential non-linearity in the time–outcome relationship. Across all pandemic phases, spline-based models demonstrated a significantly better fit than linear models (likelihood ratio tests, all *p* < 0.001). In the prolonged pandemic phase, the non-linear, selection-adjusted association between call-to-emergency department time and neurological outcome is illustrated in Figure 5.

Second, the stability of linear odds ratios was evaluated by excluding extreme call-to-emergency department times. During the prolonged pandemic phase, the direction of the association reversed after excluding the upper 5% of time values (OR 0.90; 95% CI, 0.85–0.95), suggesting that the apparent protective effect was driven by the distributional tail (Supplementary Table S1).

Third, to explicitly assess selection bias, we compared the time-valid cohort with excluded patients (Supplementary Table S2). Patients excluded due to missing time data had significantly lower rates of witnessed arrest (10.9% vs. 100%), shockable rhythms (7.2% vs. 18.6%), and good neurological outcome (2.0% vs. 8.0%), confirming non-random exclusion of poor-prognosis cases.

Fourth, to account for this bias and potential residual confounding, we employed inverse probability weighting (IPW) and performed a fully adjusted analysis including bystander CPR, place of arrest, and comorbidities (hypertension, diabetes, heart disease). As shown in Supplementary Table S1, the reversal pattern remained robust even after full adjustment (OR 1.08; 95% CI, 1.05–1.11) and IPW application.

Taken together, these sensitivity analyses indicate that the apparent phase-specific transformation of the time–outcome association is driven by non-linearity and cohort selection structure, rather than representing a robust protective effect of prolonged prehospital time.

Predicted probabilities of good neurological outcome (CPC 1–2) were estimated using a restricted cubic spline model (df = 3). Shaded areas indicate 95% confidence intervals. Predictions are shown for a reference patient (median age, male sex, shockable rhythm). IPW: inverse probability weighting (applied to adjust for selection bias arising from the exclusion of patients with missing prehospital time data).

## 4. Discussion

In this nationwide registry-based study, we have demonstrated that the population of out-of-hospital cardiac arrest (OHCA) patients with analyzable prehospital time data—the time-valid cohort—underwent substantial structural reconfiguration during the COVID-19 pandemic. This reconfiguration was accompanied by a stage-specific transformation of the association between call-to-emergency department (ER) time and neurological outcome. These findings suggest that the changes in prehospital time–outcome relationships observed during the pandemic cannot be interpreted as the effect of system delay alone, but should instead be understood within the broader context of cohort composition and selective inclusion.

### 4.1. Structural Reconfiguration of the Time-Valid Cohort

A central finding of this study is that the time-valid cohort was not a static or neutral analytic subset. As illustrated in Figure 2, the cohort underwent progressive structural transformation across pandemic phases, characterized by increasing patient age, declining proportion of shockable rhythm, increasing bystander CPR, and worsening neurological outcomes.

#### 4.1.1. Age Shift and Selective Cohort Entry

The median age of the time-valid cohort increased from 70 years pre-pandemic to 72 years during the prolonged pandemic period (Figure 2C). This shift likely reflects differential selection into the time-valid cohort rather than a true population-level aging of OHCA patients. During the pandemic, younger patients may have been more likely to experience arrests in settings where time documentation was incomplete or chaotic—such as outdoor locations, workplaces, or social gatherings—whereas older patients’ arrests more commonly occurred in structured care environments (homes with family present, nursing facilities) where arrest recognition time could be reliably documented [18,19]. Furthermore, pandemic-related delays in seeking care for acute cardiac symptoms disproportionately affected younger patients who may have avoided hospitals due to infection concerns, potentially leading to more unwitnessed or poorly documented arrests in this population [20].

#### 4.1.2. Declining Shockable Rhythm Despite Increased Bystander CPR

The proportion of shockable initial rhythms declined steadily from 21.7% pre-pandemic to 17.5% during the prolonged pandemic period (Figure 2A), a finding consistent with reports from multiple international studies [7,8,9,10,12,13,21]. Several mechanisms may explain this observation. First, the aging of the cohort itself contributes to lower shockable rhythm rates, as older patients more commonly present with asystole or pulseless electrical activity due to underlying conduction system disease and non-arrhythmic arrest etiologies [22,23]. Second, the increase in medical etiology arrests during the pandemic (Table 2)—likely including respiratory failure-related cardiac arrests from COVID-19 and deferred care for chronic conditions—would be expected to manifest predominantly as non-shockable rhythms [12,24]. Third, pandemic-related hesitancy and delays in activating EMS, even when bystanders were present, may have prolonged no-flow time sufficiently for initial ventricular fibrillation to degrade to asystole before EMS arrival [25].

Notably, the increase in bystander CPR from 32.0% to 37.0% (Figure 2B) did not translate into improved outcomes (Figure 2D). This pattern has been observed in other pandemic-era studies [6,8] and may reflect several factors: (1) bystander CPR quality may have been suboptimal due to infection-related hesitancy to perform mouth-to-mouth ventilation [26]; (2) delayed EMS arrival diluted the benefit of bystander CPR [27]; (3) the patient population receiving bystander CPR during the pandemic was inherently less salvageable due to non-cardiac arrest etiologies [28]; and (4) hospital-level factors, including saturation of intensive care resources, may have attenuated survival benefits that would otherwise accrue from effective prehospital resuscitation [29,30].

#### 4.1.3. Implications for Interpreting Pandemic OHCA Studies

The structural changes demonstrated in Figure 2 have implications for the interpretation of time–outcome analyses. Prior pandemic-related OHCA studies have largely focused on comparing outcomes before and during the pandemic or quantifying changes in specific system metrics [7,8,9,10,11,12,13,14]. However, these approaches implicitly assume that the analytic population remains comparable across periods. Our findings challenge this assumption by demonstrating that even within the subset of patients with complete time data—typically considered a methodologically “clean” cohort—substantial compositional shifts occurred. Failure to account for such shifts risks attributing outcome differences to system performance alone when they may in fact reflect changes in the underlying patient population.

### 4.2. Stage-Specific Transformation of the Time–Outcome Association (Table 3)

The association between call-to-ER time and neurological outcome exhibited a distinct phase-specific pattern (Table 3). Before the pandemic, longer prehospital time was associated with worse neurological outcomes (OR 0.85 per 10-min increase), consistent with established OHCA physiology and the well-documented importance of minimizing ischemic time [4,5,6]. During the early pandemic phase, this association was attenuated (OR 0.95) and no longer statistically significant. In the prolonged pandemic phase, we observed a statistically significant reversal, with longer prehospital time paradoxically associated with better neurological outcomes (OR 1.07).

#### 4.2.1. Mechanisms Underlying the Reversal

This counterintuitive finding should not be interpreted as evidence that prolonged prehospital time confers a protective effect. Rather, we propose that this apparent reversal reflects multiple selection phenomena that altered the meaning of prehospital time as an analytic variable during the pandemic.

*Survivorship bias and selective documentation.* While Andersen and colleagues described “resuscitation time bias” as introducing bias toward apparent harm [31], we observed an inverse phenomenon consistent with survivorship bias. During the prolonged pandemic phase, patients who survived long enough to have extended prehospital times documented likely represented a physiologically resilient subgroup. Conversely, more vulnerable patients—those with severe hypoxemia or massive myocardial infarction—may have died rapidly or had their resuscitation terminated early, leading to missing time data and exclusion from the cohort [32,33]. Consequently, “long prehospital time” paradoxically served as a marker for patient resilience (survival) rather than system inefficiency.

*Hospital saturation and dilution of early arrival benefit.* During the prolonged pandemic phase, hospital systems in Republic of Korea, as elsewhere, experienced sustained strain from COVID-19 hospitalizations [32,34]. Under these conditions, the traditional advantage of rapid hospital arrival may have been attenuated. Patients arriving quickly at saturated emergency departments may have faced delays in definitive care initiation—including advanced airway management, post-resuscitation intensive care, and coronary intervention—that effectively negated the benefits of short transport time [35,36]. Conversely, patients with extended prehospital times who achieved ROSC in the field may have arrived in more stable condition.

*Extended field resuscitation as treatment rather than delay.* The increase in bystander CPR during the pandemic (Figure 2B) suggests that a greater proportion of patients received sustained chest compressions before EMS departure. For patients who achieved field ROSC, longer scene times may reflect effective resuscitation rather than system delay [37]. In this context, call-to-ER time during the pandemic may have captured a mixture of system delay (harmful) and effective field treatment (beneficial), with the latter component increasing in relative weight as pandemic conditions persisted.

*Differential documentation survival.* Finally, the time-valid cohort is defined by the availability of complete and logically consistent time records. During chaotic, high-acuity resuscitations—more common among severely compromised patients—time documentation may be incomplete or implausible, leading to exclusion from analysis [38]. Patients with longer, more controlled resuscitation efforts may have been more likely to have accurate time stamps recorded, further enriching the prolonged-time subgroup with better-prognosis cases.

#### 4.2.2. Integration with Sensitivity Analyses

Our sensitivity analyses (Supplementary Table S1, Figure 5) support these interpretations. Notably, the reversal pattern remained robust even after adjusting for additional confounders, including bystander CPR, place of arrest, hypertension, diabetes, and heart disease (OR 1.08; 95% CI, 1.05–1.11; Supplementary Table S1). However, this association was sensitive to the exclusion of extreme time values (trimming top 5%) and exhibited non-linear characteristics on spline modeling, suggesting that the phenomenon is driven by the distributional tail of resilient survivors rather than a linear protective effect.

### 4.3. Implications for Interpretation of Pandemic-Related Ohca Research

These results have implications for the interpretation of pandemic-related OHCA research. The existing literature has largely attributed worsened outcomes during the pandemic to increased EMS response or transport times, reflecting disruptions in the prehospital chain of survival [7,8,9,10,11,12,13,14]. While system delays undoubtedly occurred and contributed to outcome deterioration, our findings indicate that changes in the composition of patients included in time-based analyses may substantially influence—and potentially confound—observed associations.

Several methodological recommendations emerge from our findings:

**Explicit cohort definition.** Future studies should explicitly define and describe the characteristics of their analytic cohort, distinguishing between all OHCA patients and those with complete time data.
**Structural comparability assessment.** Analyses spanning pandemic phases should assess whether key prognostic factors (age, initial rhythm, arrest etiology) remained stable across periods, and interpret time–outcome associations in light of any observed shifts.
**Caution in causal inference.** Observed associations between prehospital time and outcome during periods of system disruption should be interpreted as potentially reflecting cohort selection rather than direct causal effects of time delay.
**Consideration of non-linearity and interaction.** As demonstrated by our spline and sensitivity analyses, linear models may obscure important non-linear relationships and phase-specific effect modification during pandemic conditions.

### 4.4. Strengths and Limitations

This study has several strengths. First, we provided a quantitative assessment of selection bias by comparing the time-valid cohort with excluded patients (Supplementary Table S2), offering empirical data on how missing records can skew the analytic population. Second, we applied multiple analytical approaches—including restricted cubic splines, inverse probability weighting (IPW), and trimming of extreme values—to explore the mechanisms underlying the “reversal” phenomenon and to address potential survivor selection. Third, the use of a large, nationwide registry allowed for the analysis of trends across distinct pandemic phases with sufficient sample size. Finally, the application of strict time-validity criteria restricted the analysis to witnessed arrests, which helped minimize confounding related to unwitnessed downtime.

This study has several limitations that must be considered. First, and most importantly, the exclusion of cases with missing or implausible time data introduces inherent selection bias. As demonstrated in our comparison of included versus excluded patients (Supplementary Table S2), data missingness was not random but was strongly associated with unwitnessed arrests, non-shockable rhythms, and poor outcomes. This confirms that the time-valid cohort is “biased by design,” representing a selected subgroup with more favorable prognostic features than the general OHCA population. Although we applied IPW to mitigate this, IPW relies on observed covariates and cannot correct for unmeasured factors such as on-scene chaos or paramedic stress that may have contributed to data loss.

Second, potential residual confounding remains. While we adjusted for key demographic and arrest characteristics, our registry lacked granular data on real-time hospital bed saturation, emergency department diversion status, or confirmed COVID-19 diagnosis for all patients. These unmeasured systemic factors could have independently influenced both prehospital times and patient outcomes.

Third, defining pandemic phases based on calendar years may introduce an ecological fallacy. By averaging outcomes over year-long periods (e.g., the prolonged phase of 2021–2022), our analysis may have diluted the acute effects of specific infection waves or short-term surges in healthcare demand.

Fourth, our primary exposure variable, call-to-ER time, is a composite interval. Due to data limitations in the excluded population, we could not reliably dissect this interval into its components (response time, scene time, and transport time) to determine which specific phase drove the observed associations.

Finally, our findings are based on a nationwide registry from Republic of Korea, which operates a single-tiered, scoop-and-run EMS system with a high-volume, centralized structure. Consequently, the specific patterns of cohort reconfiguration observed here may not be directly generalizable to regions with different EMS models or pandemic response protocols. However, the methodological implication—that system stress leads to non-random data loss and cohort bias—is likely applicable to other settings.

### 4.5. Future Directions

Future research should incorporate dynamic cohort definitions into prehospital time analyses and develop methods to quantify and adjust for selection effects during system disruptions. Comparative analyses across different EMS systems and healthcare infrastructures may further clarify how structural reconfiguration influences observed outcomes during crises. Investigation of patient-level factors that determine inclusion in time-valid cohorts—and how these factors shift during pandemics—could inform more robust analytic approaches. Finally, linkage of prehospital data with detailed in-hospital treatment and outcome data would enable more comprehensive modeling of the full care pathway during periods of system strain.

## 5. Conclusions

In this nationwide observational study, we demonstrated that the time-valid cohort of out-of-hospital cardiac arrest patients underwent substantial structural reconfiguration during the COVID-19 pandemic, characterized by progressive aging, declining shockable rhythms, and paradoxically ineffective increases in bystander CPR.

The association between call-to-emergency department time and neurological outcome reversed from adverse before the pandemic to apparently protective during the prolonged pandemic phase. This reversal likely reflects selection phenomena—including survivor bias, hospital saturation effects, and differential documentation—rather than any true benefit of prolonged prehospital time.

These findings indicate that the time-valid cohort is not a neutral analytic subset but a selectively defined population. Future pandemic-related OHCA research should explicitly assess cohort composition and exercise caution in attributing time–outcome associations to system delay alone.

## Figures and Tables

**Table 1:** Baseline characteristics of the study population by pandemic phase.

Variable	Overall (*n* = 203,893)	Pre-Pandemic (*n* = 108,457)	Early Pandemic (*n* = 30,073)	Prolonged Pandemic (*n* = 65,363)	*p* Value
Age, median (IQR) †	70 (56–81)	69 (55–80)	71 (56–81)	71 (57–82)	<0.001
Male sex, *n* (%)	130,885 (64.2)	70,049 (64.6)	19,215 (63.9)	41,621 (63.7)	<0.001
Witnessed arrest, *n* (%)	101,778 (53.1)	52,281 (52.2)	15,007 (52.5)	34,490 (54.6)	<0.001
Public location, *n* (%)	36,758 (22.5)	21,072 (24.0)	5134 (21.7)	10,552 (20.3)	<0.001
Medical etiology, *n* (%)	156,515 (76.8)	81,930 (75.5)	23,540 (78.3)	51,045 (78.1)	<0.001
Bystander CPR, *n* (%)	66,725 (32.7)	31,767 (29.3)	10,291 (34.2)	24,667 (37.7)	<0.001
Hypertension, *n* (%)	62,726 (30.8)	31,249 (28.8)	9144 (30.4)	22,333 (34.2)	<0.001
Diabetes mellitus, *n* (%)	41,928 (20.6)	20,386 (18.8)	6226 (20.7)	15,316 (23.4)	<0.001
Heart disease, *n* (%)	30,520 (15.0)	15,271 (14.1)	4632 (15.4)	10,617 (16.2)	<0.001
Shockable rhythm, *n* (%)	25,095 (12.3)	14,241 (13.1)	3590 (11.9)	7264 (11.1)	<0.001
ROSC, *n* (%)	67,859 (33.3)	35,979 (33.2)	9772 (32.5)	22,108 (33.8)	<0.001
Survival to discharge, *n* (%)	24,287 (11.9)	14,040 (12.9)	3361 (11.2)	6886 (10.5)	<0.001
Good neurological outcome, *n* (%)	9522 (4.7)	5457 (5.0)	1294 (4.3)	2771 (4.2)	<0.001

**Table 2:** Characteristics of the time-valid cohort by pandemic phase.

Variable	Overall (*n* = 62,240)	Pre-Pandemic (*n* = 31,440)	Early Pandemic (*n* = 9386)	Prolonged Pandemic (*n* = 21,414)
Age, median [IQR], years	71 [57–81]	70 [55–80]	72 [57–81]	72 [58–82]
Male sex, *n* (%)	40,255 (64.7)	20,510 (65.2)	6052 (64.5)	13,693 (63.9)
Public location, *n* (%)	13,476 (27.8)	7232 (28.9)	1880 (26.3)	4364 (26.8)
Medical cause, *n* (%)	50,182 (80.6)	25,252 (80.3)	7807 (83.2)	17,123 (80.0)
Bystander CPR, *n* (%)	21,248 (34.1)	10,066 (32.0)	3250 (34.6)	7932 (37.0)
Hypertension, *n* (%)	21,238 (34.1)	10,319 (32.8)	3131 (33.4)	7788 (36.4)
Diabetes mellitus, *n* (%)	13,572 (21.8)	6403 (20.4)	2023 (21.6)	5146 (24.0)
Heart disease, *n* (%)	10,782 (17.3)	5181 (16.5)	1669 (17.8)	3932 (18.4)
Shockable rhythm, *n* (%)	12,319 (19.8)	6815 (21.7)	1766 (18.8)	3738 (17.5)
ROSC, *n* (%)	26,678 (42.9)	13,885 (44.2)	3792 (40.4)	9001 (42.0)
Survival to discharge, *n* (%)	10,456 (16.8)	5941 (18.9)	1454 (15.5)	3061 (14.3)
Good neurological outcome (CPC 1–2), *n* (%)	5047 (8.1)	2920 (9.3)	672 (7.2)	1455 (6.8)

**Table 3:** Phase-specific associations between call-to-ER time and good neurological outcome.

Pandemic Phase	Odds Ratio	95% CI	*p* Value
Pre-pandemic (2016–2019)	0.85	0.83–0.88	<0.001
Early pandemic (2020)	0.95	0.91–1.00	0.067
Prolonged pandemic (2021–2022)	1.07	1.04–1.10	<0.001

## Data Availability

The datasets presented in this article are not readily available because the data are owned and managed by a third party, the Korea Disease Control and Prevention Agency (KDCA). Requests to access the datasets should be directed to the KDCA (https://www.kdca.go.kr).

## Supplementary Materials

The following supporting information can be downloaded at: https://www.mdpi.com/article/10.3390/medicina62020302/s1, Table S1. Sensitivity analyses for the association between call-to-emergency department time and good neurological outcome during the prolonged pandemic phase; Table S2. Comparison of baseline characteristics and outcomes between the time-valid cohort and excluded patients.
