# Supplementary material for: Structural Reconfiguration of the Time-Valid Cohort and Stage-Specific Reversal of Prehospital Time–Outcome Associations During the COVID-19 Pandemic
Source: Medicina (Kaunas). 2026 Feb 2;62(2):302. doi: 10.3390/medicina62020302 (PMC12941986; doi:10.3390/medicina62020302)
Supplement: Supplementary file 1 [file medicina-62-00302-s001.zip › medicina-4120636-supplementary.pdf]

Table S1. Sensitivity analyses for the association between call-to-emergency department time and good neurological outcome during the prolonged pandemic phase

Analysis	Model specification	OR per 10-min increase (95% CI)	p value	Interpretation
Primary (unweighted)	Logistic, linear	1.07 (1.04–1.10)	<0.001	Linear summary
Trimmed (95%)	Logistic, linear; upper 5% excluded	0.90 (0.85–0.95)	<0.001	Direction reversal
Trimmed (99%)	Logistic, linear; upper 1% excluded	1.01 (0.98–1.05)	0.47	Non-significant
IPW-adjusted	Logistic, linear; IPW	1.10 (1.06–1.14)	<0.001	Selection-adjusted
Fully Adjusted	Logistic, linear;<br>Adj: Primary + B-CPR, Place, Comorb*	1.08 (1.05–1.11)	<0.001	Confounder-robust
Spline (IPW)	RCS (df=3); IPW	—	<0.001*	Non-linearity (LRT)

*Likelihood ratio test comparing spline vs linear model.

Adj: Adjusted for age, sex, initial rhythm, bystander CPR, place of arrest, hypertension, diabetes, and heart disease.

Estimates vary across trimming thresholds, indicating sensitivity of linear summaries to distributional tails. Spline analyses demonstrate significant non-linearity.

Table S2. Comparison of baseline characteristics and outcomes between the time-valid cohort and excluded patients

Variable	Overall (n=203,893)	Excluded (Missing Data) (n=113,171)	Included (Time-valid) (n=90,722)	SMD*	p value
Age, median [IQR]	70 [56, 81]	70 [55, 80]	71 [57, 81]	0.074	<0.001
Male sex, n (%)	130,885 (64.2)	72,034 (63.7)	58,851 (64.9)	0.025	<0.001
Witnessed arrest, n (%)	101,778 (53.1)	11,056 (10.9)	90,722 (100.0)	4.035	<0.001
Shockable rhythm, n (%)	25,095 (12.3)	8,190 (7.2)	16,905 (18.6)	0.345	<0.001
ROSC, n (%)	67,859 (33.3)	27,162 (24.0)	40,697 (44.9)	0.450	<0.001
Survival to discharge, n (%)	24,287 (11.9)	8,564 (7.6)	15,723 (17.3)	0.299	<0.001
Good neuro outcome, n (%)	9,522 (4.7)	2,252 (2.0)	7,270 (8.0)	0.279	<0.001

* SMD: Standardized Mean Difference. p-values calculated using chi-square test for categorical variables and Wilcoxon rank-sum test for continuous variables.
